# Editorial: Plant growth-promoting bacteria as a key tool for future agriculture: Agronomic, molecular and omics approaches

**DOI:** 10.3389/fmicb.2023.1168891

**Published:** 2023-03-17

**Authors:** Clara Ivette Rincón-Molina, Víctor Manuel Ruíz-Valdiviezo, Reiner Rincón-Rosales, José David Flores Félix

**Affiliations:** ^1^Instituto Tecnológico de Tuxtla Gutiérrez/Tecnológico Nacional de Mexico, Tuxtla Gutiérrez, Mexico; ^2^CICS-UBI - Health Sciences Research Centre, University of Beira Interior, Covilhã, Portugal

**Keywords:** plant growth-promoting bacteria, rhizosphere, biostimulant, omics, biofertilizer

Plant growth-promoting bacteria (PGPB) are a fundamental tool in the agriculture of the future because the mechanisms they exhibit are key in improving agricultural efficiency, increasing the integration of agriculture into the environment, and reducing the ecological footprint of agriculture (Bizos et al., 2020). These bacteria have shown to be capable of interacting at different levels with crops, both through direct mechanisms, such as nitrogen fixation or phosphate solubilization, and indirect mechanisms, such as induction of systemic resistance (ISR) or competition for space through the formation of biofilms (Haskett et al., 2020). In recent years, and through the incorporation of omics techniques (genomics, transcriptomics, proteomics, ionomics, and metabolomics), we are starting to understand the depth of plant-microorganism interactions and define a holobiont with the implications that this has for design strategies in agriculture (Riva et al., 2022). These new approaches have shown that an in-depth study of the interactions between PGPB and crops is necessary to understand how to modulate and direct nutrient acquisition and plant improvement to biotic and abiotic stresses through the application of PGPB. The main objective of this Research Topic is to elucidate plant-microorganism interactions through the application of omic techniques and the use of multidisciplinary approaches. Within this topic, 14 articles have been published, providing a deeper understanding of plant-microorganism interactions in agricultural systems and the implications for improving crop productivity.

Nordstedt and Jones studied the potential of *Serratia plymuthica* MBSA-MJ1 as a PGPB, showing the usefulness of genomic analysis and the ability of this strain to improve the development and vigor of flower crops of *Petunia* × *hybrida* (petunia), *Impatiens walleriana* (impatiens), and *Viola* × *wittrockiana* (pansy), as well as increased root, shoot, and leaf development, and nutrient absorption. Dobrzyński et al. also evaluated the capacity of the *Bacillus pumilus* species through the study of the strains *B. pumilus* W8, *B. pumilus* LZP02, *B. pumilus* JPVS11, *B. pumilus* TUAT-1, *B. pumilus* TRS-3, and *B. pumilus* EU927414 for better plant production. The authors observed improvements in vegetative parameters, substance content (amino acids, proteins, and fatty acids), and oxidative enzymes. Some strains also showed an important capacity to modulate the rhizosphere of the hosts. On the other hand, the study of the rhizosphere, whose diversity is not yet known, is essential in the search for new inoculants. Li et al. described *Paenibacillus monticola*, a novel bacteria that presented important capacities to promote development in *Arabidopsis* and *Trifolium repens*.

Ni et al. evaluated the capacity of the consortium of *Aspergillus niger* MJ1, *Pseudomonas stutzeri* DSM4166, and *P. fluorescens* CHA0-nif mutant strain on the quality of lettuce and cucumber. The authors discovered a positive effect on the production of these vegetables and modulation of the associated microbial populations to these crops, with enrichment in *Pyrinomonadaceae* and *Blastocatellia*. The application of PGPB in agriculture is a tool to improve crop efficiency, as shown by Yu et al. through the application of *Pseudomonas* sp. JP233, a phosphate solubilizing bacterium, which improved the absorption of this nutrient in corn without increasing P leaching.

The inoculation of certain PGPB strains can have a decisive effect on the rhizosphere, such as *Serratia marcenscens* X-45, whose inoculation not only improves vegetative and productive parameters in *Indigofera pseudotinctoria*, but also increases the abundance of *Bradyrhizobium*, an endosymbiont of this legume, from 1 to 42% in its rhizosphere, as demonstrated by Zheng et al.. This work strategy was also used by Deng et al. to study *in silico* the potential of *B. aryabhattai* LAD as a PGPB, identifying the main genes involved in growth promotion mechanisms and correlating with improvements in maize development.

Chai et al. studied the assessment method of PGPB, determining that due to the production of lipopeptidoglycans, as well as the need to adapt the inoculation method to the culture, Gram-positive bacteria are less sensitive to the adhesion method used than Gram-negative bacteria. The use of vermicompost and millicompost in conjunction with *Bradyrhizobium* sp. was studied by da Silva et al.. The authors showed an increase in taxa belonging to *Sphingobacteriaceae, Chitinophaga*, and *Actinobacteria*, thus enrichment in *Erysiphe diffusa* and *Thanatephorus cucumeris* related to phytopathogens. However, no disease was observed in soybean plants.

Devarajan et al. evaluated the use of statistical data integration methods, namely, principal component analysis (PCA) of concatenated data, multiple co-inertia analysis (MCIA), and multiple kernel learning (MKL), to improve PGPB selection. They demonstrated that data integration methods could complement the single-table data analysis approach and provide better insight into the microbial strain selection process.

The complexity of plant-microorganism interactions is proving to be significant, with elements such as enzymes (i.e., serine protease Sp1 from *B. licheniformis)* involved in biocontrol processes being capable of inducing upregulation of 150 differentially expressed genes (DEGs) and downregulation of 209 DEGs by RNA-seq technology. Yang et al. demonstrated that new application strategies for bacterial biofertilizers or their derivatives are possible. Another aspect studied in recent years is the role of volatile organic compounds (VOCs) in promoting plant growth and plant health. He et al. showed that the production of VOCs by *Streptomyces* sp. TOR3209 increases the expression of genes related to the improvement of plant development, such as UDP-glycosyltransferase or glutamate receptors.

In addition, not only are applied assays precise but the use of metatranscriptomic techniques allowed for the proteomic study of symbiosomes in *Phaseolus vulgaris* inoculated with *Rhizobium etli* CFN42. Taboada-Castro et al. showed differences in the production of isoenzymes associated with the development conditions of the endosymbiont, allowing us to understand the transcriptional regulations and the design of inoculation strategies.

Regarding the application of treatments to improve crop adaptation, Xiao et al. demonstrated that foliar application of melatonin induced transcriptomic modification on soybean leaves and changed rhizosphere microbial community species but not in alpha diversity, which is different from urea treatment. Their research indicates how foliar application of melatonin might influence soybean yields.

In conclusion, the study of the potential of new biofertilizers is essential for the development of biofertilizers that improve the efficiency of agriculture through *in silico* and in planta studies. This aspect should be complemented by a transcriptional study to understand and clarify the plant promotion mechanisms used by these bacteria and whose knowledge will allow the establishment of new bacterial biostimulation strategies.

## Author contributions

CR-M and VR-V wrote the first draft of the manuscript. RR-R and JF revised the manuscript and wrote the final version of the manuscript. All authors contributed to the article and approved the submitted version.
